# Neuroglobin: A New Possible Marker of Estrogen-Responsive Breast Cancer

**DOI:** 10.3390/cells10081986

**Published:** 2021-08-05

**Authors:** Virginia Solar Fernandez, Marco Fiocchetti, Manuela Cipolletti, Marco Segatto, Paolo Cercola, Annalisa Massari, Sabrina Ghinassi, Francesco Cavaliere, Maria Marino

**Affiliations:** 1 Department of Science, University Roma Tre, Viale Guglielmo Marconi 446, I-00146 Roma, Italy; virginia.solarfernandez@uniroma3.it (V.S.F.); manuela.cipolletti@uniroma3.it (M.C.); 2 Department of Biosciences and Territory, University of Molise, Contrada Fonte Lappone, 86090 Pesche (IS), Italy; marco.segatto@unimol.it; 3 Division of Senology, Belcolle Hospital, Str. Sammartinese, 01100 Viterbo, Italy; paolo.cercola@asl.vt.it (P.C.); annalisa.massari@asl.vt.it (A.M.); sabrinaghinassi@yahoo.it (S.G.); francesco.cavaliere@asl.vt.it (F.C.)

**Keywords:** AKT, breast cancer survival, ductal infiltrating adenocarcinoma, estrogen receptor α, neuroglobin, tumor microenvironment

## Abstract

The expression of the α-subtype of Estrogen Receptor (ERα) characterizes most breast cancers (more than 75%), for which endocrine therapy is the mainstay for their treatment. However, a high percentage of ERα+ breast cancers are de novo or acquired resistance to endocrine therapy, and the definition of new targets for improving therapeutic interventions and the prediction of treatment response is demanding. Our previous data identified the ERα/AKT/neuroglobin (NGB) pathway as a common pro-survival process activated in different ERα breast cancer cell lines. However, no in vivo association between the globin and the malignity of breast cancer has yet been done. Here, we evaluated the levels and localization of NGB in ERα+ breast ductal carcinoma tissue of different grades derived from pre-and post-menopausal patients. The results indicate a strong association between NGB accumulation, ERα, AKT activation, and the G3 grade, while no association with the menopausal state has been evidenced. Analyses of the data set (e.g., GOBO) strengthen the idea that NGB accumulation could be linked to tumor cell aggressiveness (high grade) and resistance to treatment. These data support the view that NGB accumulation, mainly related to ER expression and tumor grade, represents a compensatory process, which allows cancer cells to survive in an unfavorable environment.

## 1. Introduction

Female breast cancer represents the most commonly diagnosed cancer among both sexes, with an estimated 2.3 million (11.3% of total) new cases in 2020 [1]. Estrogen-receptor-α-positive (ERα+) breast cancers represent 79% of the reported cases worldwide [2]. They refer to the expression of ERα, the activation of which by 17β-Estradiol (E2) plays a pivotal role in inducing breast cancer cells’ proliferation and survival [3,4]. Consistent with ERα activities in the development, progression, and treatment of breast cancer, the endocrine therapy is used in clinical practice to interfere with cancer cell growth and survival through the inhibition of E2 synthesis (aromatase inhibitors) or the antagonism of ERα activation (e.g., fulvestrant, tamoxifen) [4,5]. However, a high percentage of ERα+ breast cancers are de-novo (15–20%) or acquired resistance (30–40%) to endocrine therapy progressing from hormone dependence to independence, although a functional ERα pathway is still maintained [4,6,7]. Therefore, basic studies shifted their interests on molecular mechanisms and mediators of ERα action to find alternative targets and new possible biomarkers to improve therapeutic interventions and the prediction of treatment response [6]. 

In such a context, we found a new compensatory protein named neuroglobin (NGB), which is up-regulated and re-allocated to mitochondria by E2 in ERα+ breast cancer cells [8]. Since its discovery in neurons, NGB has attracted great research interest, in particular, because of its cytoprotective effect against several types of insults [9,10,11,12,13,14]. Consistent with this, our previous data define NGB accumulation as the key factor in the E2-activated ERα pathway devoted to breast cancer cell survival against oxidative stress and chemotherapeutic agents [8,15]. In addition, our recent findings further identified the ERα/AKT/NGB pathway as a common pro-survival process activated in different, ERα-positive, breast cancer cells [16]. 

Notwithstanding this, the possible association between high levels of NGB and human cancer progression is still unclear and strongly debated [17,18,19]. On one hand, the enhanced expression of NGB in cancer cells and in the primary tumor of brain and non-brain origins (e.g., breast, lung) has been demonstrated and connected to the endogenous stress-related mechanism of cancer defense [17,19]. On other hand, a tumor suppression function for NGB accumulation has been proposed [20]. Finally, no expression of *NGB* mRNA in non-nervous tumors and normal organs [18] has been reported. These conflicting data, obtained with dissimilar approaches in different cancer tissues, could imply that a cellular/tissue context-dependent effect of NGB exists, highlighting the need to verify in vivo the possible connection between NGB accumulation and breast cancer progression. Here, the hypothesis that E2-induced NGB accumulation is a conserved compensatory mechanism important for breast cancer progression, by averting mitochondrial apoptosis, has been evaluated in ERα-expressing breast ductal carcinoma tissue samples with different grades derived from patients who were not subjected to chemotherapy or endocrine treatments earlier. 

## 2. Materials and Methods

### 2.1. Reagents

Protease inhibitor cocktail, bovine serum albumin fraction V (BSA), phosphatase inhibitor cocktail, PBS, Tris buffer, anti-vinculin (clone hVIN-1, mouse monoclonal) anti-Neuroglobin (clone 6G1.1, mouse monoclonal) were purchased from MERCK (Darmstadt, Germany), and polyclonal anti-NGB antibody from Santa Cruz Biotechnology (FL-151, Santa Cruz, CA, USA). Bradford protein assay was obtained from Bio-Rad Laboratories (Hercules, CA, USA). The anti-phospho-ERα (pERα Ser118, 16J4 mouse monoclonal) and anti-phospho-AKT (pAKT, 193H12, rabbit monoclonal) antibodies were purchased from Cell Signalling Technology Inc. (Beverly, MA, USA). Specific antibodies against ERα (HC20, rabbit polyclonal), Estrogen Receptor β (ERβ, H-150 rabbit polyclonal), Bcl-2 (C-2, mouse monoclonal), Tumor necrosis factor receptor-associated protein 1 (TRAP-1, TR-1 mouse monoclonal), protein phosphatase 2A (PP2A, FL-309, rabbit polyclonal), G protein-coupled estrogen receptor 1 (GPER, N-15 rabbit polyclonal), and AKT (B-1, mouse monoclonal) were obtained from Santa Cruz Biotechnology (Santa Cruz, CA, USA). Chemiluminescence reagent for Western blot ECL was obtained from Bio-Rad (Hercules, CA, USA).

All the other products were obtained from MERCK. Analytical- or reagent-grade products were used without further purification.

### 2.2. Human Subjects

All followed procedures were in accordance with the Ethics Committee Lazio 1 Protocol number 2012/CE Lazio 1 and with the Helsinki Declaration of 1975 (World Medical Association Declaration of Helsinki 2000). Signed informed consent was obtained from all individual participants included in the study. Fifty-three women of different ages and types of ER-positive breast cancers (Table 1) were recruited in the study.

### 2.3. Breast Cancer Tissues

Surgical breast paraffin-embedded sections and cryo-conserved fresh tissue were collected at Belcolle Hospital in Viterbo from primary tumors and from normal tissue of patients who had undergone breast surgery. Based on the histopathological analysis of surgical specimens, we selected ERα+ ductal carcinoma divided into G2 (*n* = 38) and G3 (*n* = 15). The G1 grade or well-differentiated represents only the 7% of the breast cancers with respect to the G2 (71%, the most frequent) and G3 (22%) grades. The cells of the G1 grade are slower growing and look more like normal breast tissue. This low frequency and high differentiation led us to increase the number of G2 and G3 specimens, avoiding G1 samples. In Table 1, the considered histopathological characteristics of patients are reported. All tissue samples were stored and conserved until use at −80 °C. 

### 2.4. Breast Tissue Immunohistochemistry

**Figure 1:**
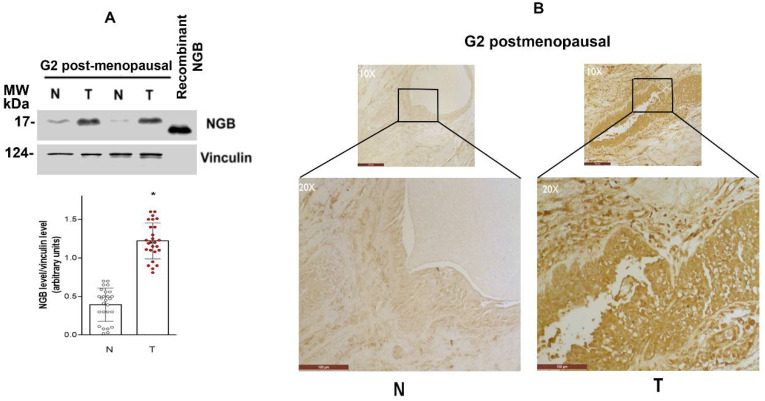
NGB levels in breast cancer tissue. Representative Western blot images of NGB protein levels (**A**) in two G2-grade post-menopausal specimens of breast cancer (T) and their normal counterpart (N). The amount of protein was normalized by comparison with vinculin levels. A total of 5 ng of recombinant NGB was used as protein standard. Data are the mean ± SD of 26 tissues. * *p* < 0.001 was calculated with Student’s t-test with respect to normal tissues. (**B**) Representative immunohistochemical staining of NGB (mouse monoclonal Clone 6G1.1 anti-NGB MERCK) in 1 out of 10 breast G2-grade post-menopausal specimens of breast cancer (T) and normal counterpart (N). The scale bar is 100 μm/cm. The black square refers to the below-reported optical magnification.

For immunohistochemistry analysis, 10 ERα+ Grade 2 infiltrating ductal carcinoma were selected. Breast cancer paraffin-embedded sections were deparaffinized in HistoChoice Clearing Agent MERCK (Darmstadt, Germany) and rehydrated in a graded series of ethanol. For antigen retrieval, the sections were boiled in a microwave in 10 mM of sodium citrate (pH 6.0) for 3 min. The sections were incubated in 3.0% H_2_O_2_ solution for 3 min to block endogenous peroxidase activity. Following a blocking step with 3% BSA in PBS + Triton-X 100 0.5%, the sections were incubated with the primary mouse monoclonal NGB (Clone 6G1.1 Merck, Figure 1 and Figure 4) o/n at 4 °C. The possibility of non-specific staining has been excluded by using a polyclonal anti-NGB antibody (FL-151, Santa Cruz, CA, USA). After washing with PBS, sections were incubated with anti-mouse secondary antibody for 30 min at room temperature. Antibody binding was detected using a ImmPACT DAB Peroxidase Substrate Kit (Vector Laboratories Inc, Burlingame, CA, USA).

### 2.5. Protein Extraction and Western Blot Assay

Tissue fragments were cut into small pieces and then homogenized in 10 vol of YY buffer (50 mM HEPES at pH 7.5, 10% glycerol, 150 mM NaCl, 1% Triton X-100, 1 mM EDTA, 1 mM EGTA) containing 0.70% (*w*/*v*) SDS, with 65 strokes in a glass-Teflon homogenizer. Homogenates were centrifuged at 10,000× *g* for 10 min. Supernatant was collected for Western blots. Total proteins were quantified using the Bradford Protein Assay. Solubilized proteins (20 μg) were resolved by 7%, 10% or 15% SDS-PAGE at 100 V for 1 h at 24.0 °C and then transferred to nitrocellulose with the Trans-Blot Turbo Transfer System (Bio-Rad, Hercules, CA, USA) for 10 or 7 min. The nitrocellulose was treated with filtered 5% (*w*/*v*) BSA in 138.0 mM NaCl, 25.0 mM Tris, pH 8.0, at 24.0 °C for 1 h and then probed overnight at 4.0 °C with either anti-NGB (mouse monoclonal Clone 6G1.1, final dilution 1:1000), anti-Bcl-2 (final dilution 1:1000), anti-pERα (final dilution 1:1000), anti-ERβ (final dilution 1:1000), anti-GPER (final dilution 1:1000) and anti-pAKT (final dilution 1:1000). The nitrocellulose was stripped by the Restore Western Blot Stripping Buffer (Pierce Chemical, Rockford, IL, USA) for 30 min at room temperature and then probed with anti-ERα (final dilution 1:1000) or anti-AKT (final dilution 1:1000) or anti-vinculin (final dilution 1:30,000) to normalize the protein loading.

### 2.6. Mitochondria Isolation

Tissue fractionation was performed using the ApoAlert™ Cell Fractionation kit (Clontech Laboratories Inc. Mountain View, CA, USA) according to the manufacturer’s instructions. Tissue fragments were cut into small pieces, washed with Wash buffer, suspended in Fractionation Buffer Mix containing DTT 1 mM and then homogenized with 65 strokes in a glass-Teflon homogenizer. Homogenate was centrifuged at 700× *g* for 10 min. The pellet was discarded and supernatant centrifuged at 10,000× *g* for 25 min. The pellet (Mitochondrial fraction) was resuspended in YY buffer (50 mM HEPES at pH 7.5, 10% glycerol, 150 mM NaCl, 1% Triton X-100, 1 mM EDTA, 1 mM EGTA) containing 4.0% (*w*/*v*) of SDS. Supernatant (cytosolic fraction) was collected in a separate tube. The protein concentration of each fraction was determined using Bradford protein assay. Lysate of each fraction was then processed for Western blot. The mitochondrial TRAP-1 and cytosolic PP2A were used as fraction purity markers.

### 2.7. Statistical Analysis

The statistical analysis was performed with ANOVA followed by Bonferroni post-test to compare multiple samples or with Student’s *t*-test to compare two diverse samples by InStat 3.10 software system for Windows. In all cases, *p* < 0.05 was considered significant.

## 3. Results

### 3.1. NGB Expression in Breast Tumors

Fifty-three Erα-positive ductal carcinoma specimens (T = tumor) were obtained from 35-80-year-old patients and divided considering menopausal state (pre/post menopause) or the tumoral grade (G2, G3, divided in 38 and 15 samples respectively) (Table 1). Samples of non-proliferative ductal cells of the same patients were used as a control (N = normal) (see the Material and Method section for details). A significant accumulation of NGB in the G2 grade of post-menopausal patients compared to the normal counterpart is reported in Figure 1A. To further confirm the high level of NGB in breast cancer tissue, paraffin-embedded sections of ERα+ invasive ductal carcinoma grade G2 from 10 breast cancer patients and their healthy counterparts were subjected to immunohistochemistry staining against NGB. The NGB immunoreactivity is barely detectable in the normal tissue, whereas cancer samples show a strong positive NGB staining in the epithelial cells (Figure 1B and Supplementary Figure S1).

### 3.2. Association between NGB and Estrogen Receptor Levels and Activities in Breast Tumors

**Figure 2:**
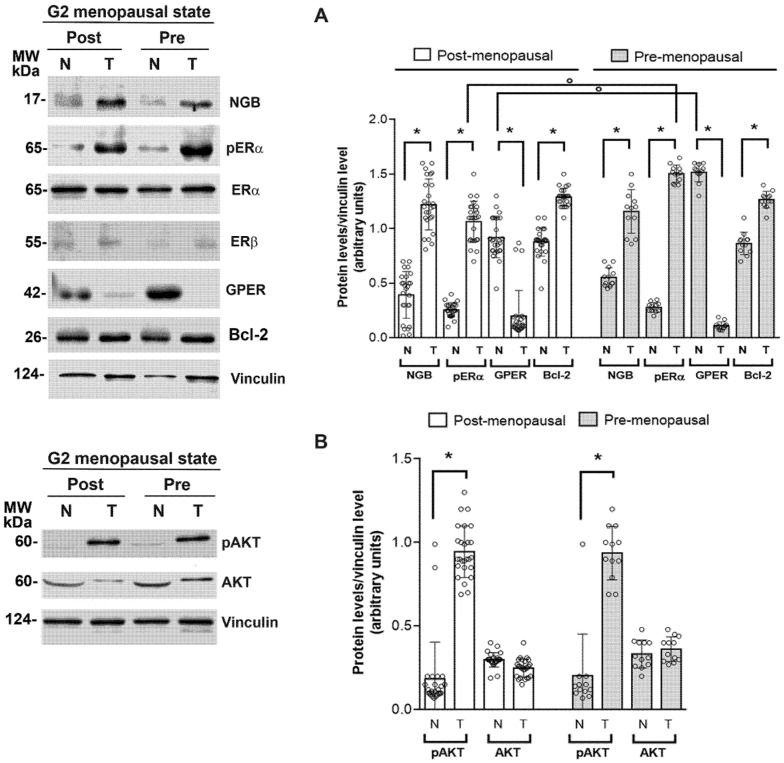
Association between NGB levels and estrogen receptors in breast cancer tissue. Representative Western blot images of NGB, pERα, ERα, ERβ, GPER, Bcl-2 protein levels (**A**) and AKT levels and activation (**B**) in G2-grade post- (*n* = 26) and pre-menopausal (*n* = 12) specimens of breast cancer (T) and their normal counterpart (N). The amount of protein was normalized by comparison with vinculin levels. Data are the mean ± SD. *p* < 0.001 was calculated with Student’s t-test with respect to the normal or tumoral (e.g., GPER) counterpart (*) and vs. the same parameter on the post-menopausal sample (°).

Clinical practice considers ERα+ breast cancers those expressing this receptor subtype in the nucleus. Although all tumor tissues selected for this study were ERα+, due to the heterogeneity of breast cancer, we would determine if the other subtype of estrogen receptor (e.g., ERβ) as well as the seven uncanonical membrane-spanning estrogen receptor (e.g., GPER), were involved in NGB accumulation in cancer tissues. In addition, the activation status of ERα (e.g., phosphorylation at Ser118, pERα), the Bcl-2 levels, an E2/ERα-dependent apoptosis associated factor [21], and the activation of AKT, one of the ERα-dependent signals important for NGB accumulation in cancer cell lines [16], have been evaluated in G2-grade tumors of pre-and post-menopausal patients. Figure 2A confirms that both pre-and post-menopausal tissues contain relevant levels of ERα, but only in cancer tissues ERα is phosphorylated in Ser118 sustaining its activated status [22,23], which is higher in pre-menopausal than in post-menopausal samples. Consistent with the activation status of ERα, a high level of Bcl-2 protein and the increased phosphorylation of AKT is reported in cancer specimens when compared to the normal counterparts (Figure 2A,B, respectively). None of the other estrogen receptors is expressed at a relevant amount in cancer tissues. Indeed, ERβ is barely detected both in normal and cancer tissues, while GPER levels are lower in cancer tissues than in their normal counterparts (Figure 2A).

In line with previous results obtained in breast cancer cells [24], high levels of NGB have been detected only in tumor samples versus their matched normal tissue, sustaining a key role of the globin in breast cancer pathophysiology. 

### 3.3. NGB Association with Tumor Grade

**Figure 3:**
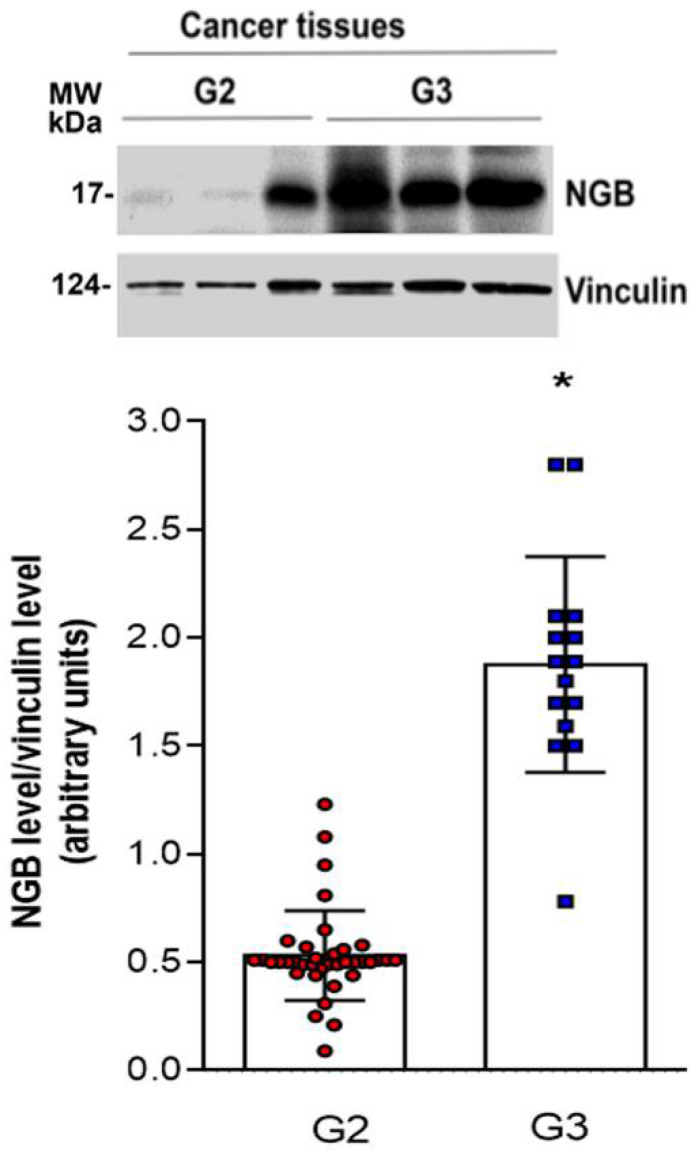
NGB protein levels in tumor samples of breast cancer patients. Representative Western blot images (upper panel) of tissue levels of NGB from G2 and G3 samples and correspondent densitometric analysis of all analyzed tissues (*n* = 38, *n* = 15, respectively) (bottom panel). The amount of protein was normalized by comparison with vinculin levels. Data are means ± SD. *p* < 0.01 was determined with ANOVA followed by Bonferroni post-test vs. G2 (*) samples.

To evaluate whether the levels of NGB could change with cancer progression, the globin expression has been assessed among tumor samples divided based on their stratification for the histological grade (G2 and G3; *n* = 38 and *n* = 15 respectively). A significantly high NGB accumulation in G3 tumor tissue, which represents the most aggressive grade with marked variability, is reported (Figure 3).

### 3.4. NGB Localization in Breast Cancer Tissues

**Figure 4:**
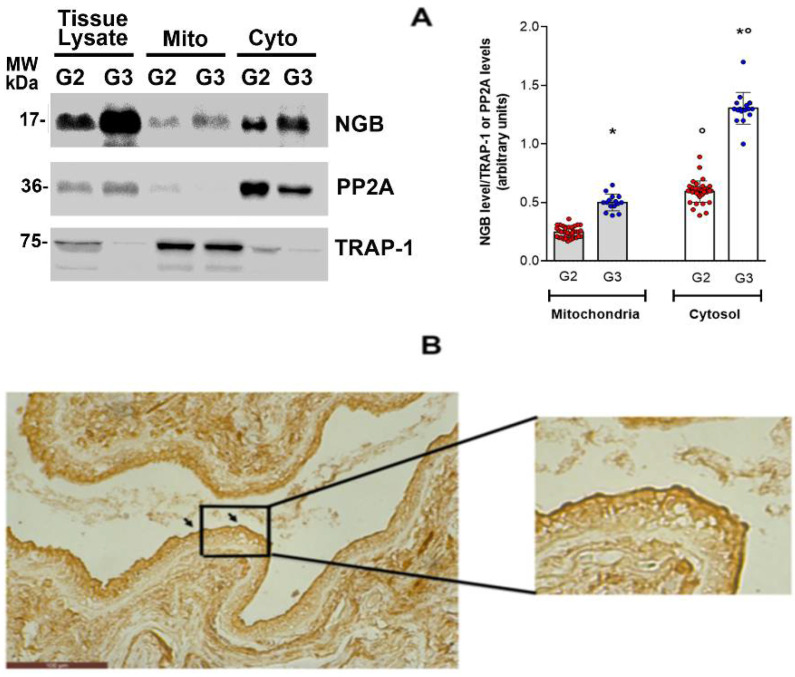
NGB localization in breast tissues. (**A**) Representative Western blot image (left panel) of NGB levels in whole tissue (Tissue lysate) and in subcellular (Mitochondria-Mito, Cytosol-Cyto) fractions of G2 and G3 samples and densitometric analysis (right panel) of NGB levels in subcellular fractions among all available tissues (G2 *n* = 38, G3 *n* = 15, respectively). The amount of protein was normalized by comparison with the cytosolic marker protein phosphatase 2A (PP2A) and the mitochondrial marker tumor necrosis factor-associated protein 1 (TRAP-1). Data are means ± SD. *p* < 0.01 was determined with ANOVA followed by Bonferroni post-test vs. G2 (*) samples and vs. correspondent mitochondrial fraction (°). (**B**) Representative image shows positive NGB staining (mouse monoclonal Clone 6G1.1 anti-NGB MERCK) in the epithelial cells of the tumoral section in a G2 ERα+ breast cancer tissue sample (total *n* = 10). The scale bar is 100 μm/cm. The black square refers to image detail reported on the right as a digital magnification.

As reported above, the increase in NGB levels induced by E2, via ERα and AKT activation, plays an anti-apoptotic effect when the globin is accumulated into the mitochondria of breast cancer cells [16]. This evidence prompted us to study the compartmentalization of NGB in the cytosolic and mitochondrial fractions of G2 and G3 samples. TNF receptor-associated protein 1 (TRAP1) and the protein phosphatase 2A (PP2A) were used as fractionation purity markers of the mitochondrial and cytosolic fraction, respectively. Due to the low levels of expressed NGB (Figure 1), no NGB reactivity was reported in the cytosolic and mitochondrial fraction of normal tissues (data not shown), whereas a significant accumulation of NGB was reported not only in the cytosol, but also in the mitochondrial fraction of cancer tissues (Figure 4A). The ductal carcinoma of G3-grade tissue showed a higher NGB immunoreactivity compared to G2 tissues in both whole lysate and subcellular fraction (Figure 4A). Thus, at least two different pools of NGB, cytosolic and mitochondrial, exist in the breast tissues and they can act to preserve cancer cell survival and resistance to environmental stresses. Moreover, immunohistochemical analysis of G2 breast cancer tissues revealed strong NGB staining near the border of the duct close to the lumen, sustaining the idea of a possible accumulation of NGB on the cell periphery (Figure 4B).

### 3.5. Effect of NGB Expression in Breast Cancer Patient Survival

**Figure 5:**
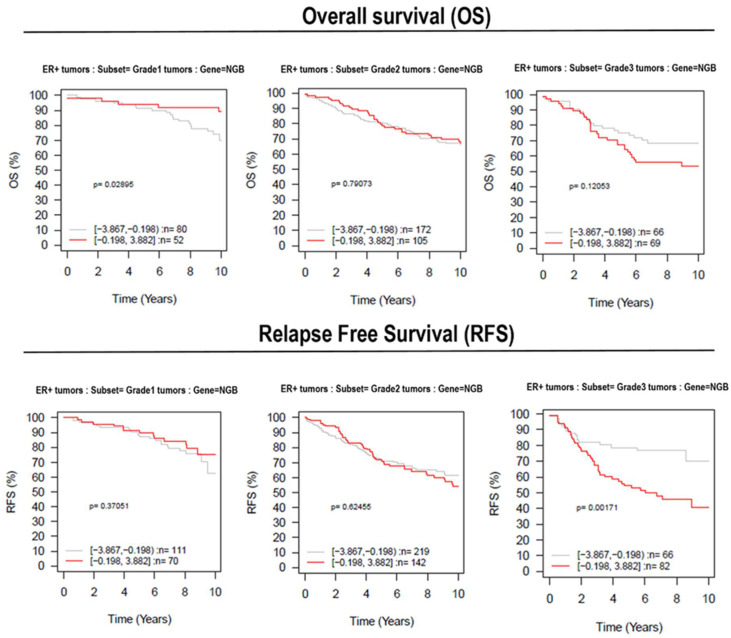
Data set analysis of survival in patients with different NGB expressions. Kaplan–Meier plots of high (red line) or low (gray line) NGB-expressing tumor samples (ID = 58157) available in the GOBO (Gene expression-based Outcome for Breast cancer Online). The data have been selected from ERα+ samples based on tumor grade (lowest at left, highest at right). The overall (upper panels) and the relapse-free (bottom panels) survival have been selected as outcomes.

The results reported here showing the association between NGB accumulation and ERα+ ductal carcinoma tissues could sustain the promise of improving prognostication and treatment decisions for breast cancer patients. However, the heterogeneity of breast cancer emphasizes the need for validation of relative gene in larger sample sets stratified into relevant subgroups. Recently, a multifunctional online tool, GOBO (http://co.bmc.lu.se/gobo. Last access 4 August 2021), has been described [25]. GOBO allows for a range of different analyses to be performed in an 1881-sample breast tumor data set, and a 51-sample breast cancer cell line set, both generated on Affymetrix U133A microarrays. By using the GOBO data set, we analyzed the possible association between *NGB* gene (ID = 58157) expression levels with Overall Survival (OS) and Relapse-Free Survival (RFS) outcomes in G1, G2, and G3 subgroups of ERα+ breast cancer data set. In Figure 5, Kaplan–Meier analysis shows positive differences regarding OS between high- or low-*NGB*-expressing tumor samples in the G1 grade, which was attenuated in G2-grade patients. Although not statistically significant, this positive difference was found to be reverted in G3-grade patients: notably, OS is lower in a high-*NGB*-expressing tumor (red line) when compared to the low-*NGB*-expressing patients after three years (gray line). Moreover, high-*NGB*-expressing tumor samples of G3 patients have a high significant (*p* = 0.001; Figure 5) lower 10-year RFS with respect to the low-expressing samples.

## 4. Discussion

Since its discovery in neurons, NGB has attracted great research interest, particularly because of its cytoprotective effects against several types of insults [26]. Although the expression of NGB has been found in both normal and cancerous extra-nervous tissues [17,19], the correlation between NGB and human cancer is still unclear and strongly debated principally because other studies affirm that no significant levels of *NGB* transcript could be detected in extra-nervous cancers and normal tissues [18]. Despite the reported discrepancies, our previous findings define NGB as an E2-inducible protein in ERα+ breast cancer cell models (MCF-7, T47D, ZR51), which accumulation is part of the E2-induced pathway devoted to the survival of breast cancer against stress injuries of a different nature [8,24]. Although cell line models are useful to define molecular candidates as markers of malignant transformation, several differences exist between cell lines and primary tumors. Indeed, the absence of any stromal and extracellular interaction and the selective pressure in cell culture could explain why several aspects of primary cancer biology are not fully represented by cancer cell systems [27]. This evidence prompted us to evaluate if the NGB accumulation reported in cancer cells is a conserved compensatory mechanism important for ERα+ ductal carcinoma progression. 

Our data indicate that NGB is greatly accumulated in all breast tumor samples analyzed with respect to their normal counterparts, where the protein levels are barely detected. Remarkably, in line with the previous data reported on cell lines [8,24], the NGB buildup positively associates with the active status of ERα (i.e., phosphorylation at Ser118) [22,23] and to the E2-inducible Bcl-2 accumulation and AKT activation [15]. Surprisingly, despite the dramatic decrease in circulating E2 in post-menopausal women, the levels of NGB did not change between pre-and post-menopausal cancer samples or in the activation of ERα or AKT. This is in line with the observation that breast cancer cells maintain an E2 concentration 10–20-fold higher than its corresponding plasma levels due to E2 uptake from surrounding tissues that include adipose tissue [28,29,30] in which the hormone synthesis occurs starting from steroidogenic precursors. The absence of any association between NGB accumulation in breast cancer tissues and other estrogen receptors (e.g., ERβ and GPER) strongly sustains the results obtained in cancer cell lines confirming the pivotal involvement of the ERα/AKT pathway in NGB accumulation in breast cancer tissues. Although the literature on isoform-specific roles of AKT in cancer progression is still debated, the three functional AKTs encoded by distinct genes are all differently involved in cancer growth and progression (AKT1), survival (AKT1 and AKT3), as well as in metastasis, migration, and invasion (AKT2) [31]. In cancer cell lines, we demonstrated that membrane-bound Erα induces the rapid and persistent activation of PI3K-AKT [32], thus leading, among other effects, to the block of NGB degradation, the increase in *NGB* gene transcription via CREB, and NGB translocation to the mitochondria, which culminate in breast cancer survival against oxidative stress and the apoptosis prevention [15,16]. The association here reported between ERα phosphorylation, AKT activation, and NGB accumulation strongly sustain that a similar pathway is also active in ERα+ ductal carcinoma tissues. 

A good association between NGB accumulation and the grades of the tumor samples was highlighted. Indeed, G3 breast cancer samples expressed significantly higher levels of the globin compared to G2 samples. G3 tumors show a lower degree of cell differentiation, a higher rate of proliferation/metastatization, and the worst outcome with respect to the other grades, which are much less aggressive [33]. The presence of high NGB levels in the highest-grade breast cancers can explain, at least in part, their bad prognosis. Indeed, since the well-known cytoprotective effect of NGB against several types of insults including oxidative stress and chemotherapy [8,15,16], it may be thought that intracellular NGB accumulation would participate in the resistance mechanisms established by cancer cells to cope against tumor micro-environmental stress, which favor the G3 cancer aggressiveness. On the other side, data reported here could define NGB as a possible new prognostic marker for the disease. Indeed, markers regarding the specific grade of breast cancer have not yet been identified to date. Our result indicates that NGB tissue analysis could represent a good biomarker index for breast-cancer-grade identification and the subsequent treatment evaluation. 

Historically, NGB accumulation in the cytosolic fraction has been associated with the regulation of several cell functions. NGB behaves as a direct scavenger of harmful excess in ROS and RNS, it acts as an upstream regulator of the intracellular signaling by activating the PI3K/AKT pathway or blocking the GDP dissociation from heterotrimeric Gα protein, and NGB can directly inhibit the apoptosome formation by reducing, through a cytosolic reaction, the cytochrome c, rendering it apoptotically inactive ([14] and therein citations). Accumulating evidence indicates that NGB is also physically and functionally associated with mitochondria. NGB overexpression preserves mitochondria ATP production, reduces ROS generation, and prevents mitochondria-mediated cell death signaling caused by physiological stressors [34]. Beyond the well-defined NGB mitochondria-associated function in neuron-derived cells, our previous data [8,24] clearly show that E2 re-localized the NGB to the mitochondrial compartment also in breast cancer cell models (MCF-7, T47D), making the globin able to exert its anti-apoptotic activities [8]. Present findings indicate, for the first time, that NGB buildup is mainly localized in the cytosol of samples, even though a significant amount of the globin is also gathered in mitochondria of tumor tissue specimens. Consistently with the high expression of NGB at a higher tumor grade, we observed an increase in NGB levels both in the mitochondrial and cytosolic compartments in G3 specimens if compared to G2. To complicate further the scenario about the functional role of NGB in breast tumors, we demonstrated that a great immunoreactivity of NGB is localized near the border of the duct close to the lumen. Recently, the co-localization of NGB with collagen I fibers at the extracellular levels has been reported in breast tissues [35], supporting the idea of an in vivo secretion of the protein to the extracellular matrix by breast cancer cells. In the future, the analysis of NGB levels in the serum of breast cancer patients, with respect to comparable healthy volunteers, could reinforce from one side the possibility that NGB can have an extracellular function and from the other that, beyond the tissue levels of NGB, the amount of serum NGB can be used as a breast cancer biomarker with a prognostic value. 

The research on the association between human cancer and globins (e.g., MB, cytoglobin, CYGB) has grown in the last years. In this context, the CYGB mRNA expression has been found downregulated in breast and lung carcinoma tissues with respect to the normal counterpart [18], and evidence suggested a tumor suppressor role for this globin [36,37]. Indeed, the overexpression and/or the induction of CYGB in breast cancer cell lines correlates with the reduction of cell migration and increased cell death [38]. Despite this, the levels of CYGB and NGB proteins were reported to generally be increased in different tumor sections in comparison to levels observed in corresponding normal tissues, leading the authors to suggest a contribution of both globins in promoting cancer survival under oxidative stress [17]. Additionally, MB expression has been reported in several epithelial cancers [39]. In breast cancer, MB levels have been correlated with luminal- ERα+ subtype and with favorable prognostic outcomes in both ERα+ and ERα- breast cancers [40]. However, contrarily to *NGB*, the GOBO analysis did not show any significant correlation between *MB* expression and Relapse-Free Survival (RFS) outcomes in the G1, G2, and G3 subgroups of the ERα+ breast cancer data set (available at http://co.bmc.lu.se/gobo. Last access 4 August 2021). In the scenario of a possible correlation between globins function in breast cancer cells, the data of an opposite link between the MB expression [41] or NGB levels ([8,15,16,35], and results reported here) and ERα activation is particularly interesting. Indeed, in line with the proposed tumor-suppressive function of MB, the levels of this protein are downregulated by the E2/ERα pathways in cell culture [41], supporting a possible MB role in counteracting the Erα-mediated function including cell growth and resistance to chemotherapeutic drugs [41,42,43]. On the other hand, the role of NGB as a target protein and mediator of the anti-apoptotic and anti-oxidant effect of the E2/ERα signaling [8,15,16,44], along with the connection between ERα activation and NGB in vivo (present paper), corroborates the idea that among the different globins, NGB could represent that involved in tumor resistance and relapse, the overexpression of which, dependently or independently from estrogen signaling, can favor cancer cell survival and, potentially, tumor aggressiveness. Present and previous results draw possible multi-point and functional intersections between the globins in human cancers whose further analysis will be necessary and could be promising for defining the role of each protein and/or the prognostic/predictive value of the “dance” of the globins across human cancers.

Overall, our results converge on the accumulation of NGB as a novel characteristic of ERα+ ductal carcinoma of Grade 3 that could be used for improving prognostication and treatment decisions for breast cancer patients. Indeed, the role played by NGB accumulation in cancer cell lines strongly sustains that this globin could increase the resilience of cancer cells against oxidative stress, nutrient deprivation, and chemotherapeutic treatment affecting the triggering of apoptosis as well as increasing cell responses to stress [8,15,24,35,45]. However, as mentioned previously, the heterogeneity of breast cancer emphasizes the need to validate a promising protein and/or its relative gene in larger sample sets stratified into relevant subgroups. Here, we used a multifunctional online tool, GOBO (http://co.bmc.lu.se/gobo. Last access 4 August 2021) [25], that allows a range of different analyses to be performed in an 1881-sample breast tumor data set. The obtained results indicate a positive correlation between *NGB* gene expression and a lower RFS in the subset of Grade 3 tumors. These data support the hypothesis that the increased expression of NGB could be linked to tumor cell resistance to treatment mainly related to ER expression and tumor grade. 

## 5. Conclusions

Taken together, these results reveal strong evidence of similarities between NGB behavior in the cancer cells’ system and primary breast cancer tissues, strengthening the idea that NGB represents a compensatory protein, which allows cancer cells to survive in an unfavorable environment. The NGB unique expression in cancer tissue, relative to the normal counterpart, its anti-apoptotic role [8], and its involvement in breast cancer insensitivity to chemotherapeutic agents (e.g., paclitaxel) [15], introduces new insights in breast cancer research, supporting the possible role of NGB as a new biomarker of breast malignant transformation and as a target for clinical interventions. Therefore, in the light of reported results that provide evidence of the existence of at least three different pools of the globin (mitochondrial, cytosolic, and extracellular), research is still needed to clarify the mechanisms underlying the mitochondrial translocation of the protein and its extra-mitochondrial function under E2 stimulation and to explore a possible NGB release and its functions.

## Figures and Tables

**Table 1:** Histopathological characteristics of tumors of patients who had undergone breast surgery.

Variable		Number of Patients (N_TOT_ = 53)	% on Total Patients
**Menopausal status**	Yes (Age ≥ 51 years)	38	71.7
	No (Age < 51 years)	15	28.3
**Hystological type**	Ductal carcinoma	53	100
**ER/PR Status**	Positive	53	100
**Grade**	G2	38 (26 Menopause Y; 12 Menopause N)	71.7
	G3	15 (12 Menopause Y; 3 Menopause N)	28.3

## Supplementary Materials

The following are available online at https://www.mdpi.com/article/10.3390/cells10081986/s1, Supplementary Figure S1 and the original images for Western blot have been uploaded with the manuscript.
