# Supplementary material for: Neuroglobin: A New Possible Marker of Estrogen-Responsive Breast Cancer
Source: Cells. 2021 Aug 5;10(8):1986. doi: 10.3390/cells10081986 (PMC8393432; doi:10.3390/cells10081986)
Supplement: Supplementary file 1 [file cells-10-01986-s001.zip › cells-1305467-supplementary.pdf]

# Supplementary Material

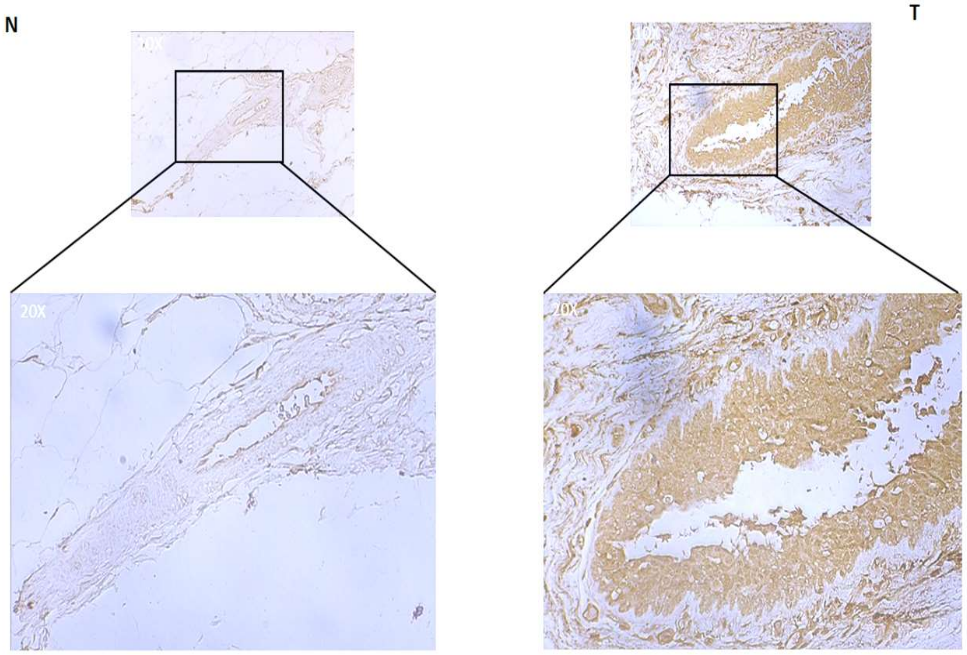


**Figure S1.** Representative immunohistochemical staining of NGB in 1 out of 10 breast G2 grade post-menopausal specimens of breast cancer (T) and normal counterpart (N) obtained by using a polyclonal anti-NGB antibody ( FL-151, Santa Cruz). The black square refers to the below reported optical magnification.
